# Opioids for acute and chronic pain when receiving psychiatric medications

**DOI:** 10.1371/journal.pone.0286179

**Published:** 2023-09-26

**Authors:** Chandrashekar Janakiram, Ilya Okunev, Eric P. Tranby, Paul Fontelo, Timothy J. Iafolla, Bruce A. Dye

**Affiliations:** 1 Amrita School of Dentistry, Amrita Vishwa Vidhyapeetham, Cochin, India; 2 National Library of Medicine, National Institute of Health, Bethesda, Maryland, United States of America; 3 Health Data Analytics Institute, Dedham, Massachusetts, United States of America; 4 Analytics and Evaluation, Care Quest Institute for Oral Health, Boston, Massachusetts, United States of America; 5 National Institute of Dental and Craniofacial Research, National Institutes of Health, Bethesda, Maryland, United States of America; 6 University of Colorado School of Dental Medicine, University of Colorado Anschutz Medical Campus, Aurora, Colorado, United States of America; University of South Australia, AUSTRALIA

## Abstract

**Background:**

People with mental health disorders (MHD) like depression and anxiety are more likely to experience substance use disorders (SUDs) than those without MHD. This study assesses opioid prescription patterns for acute or chronic pain management in patients receiving medication for depression and/or anxiety.

**Methods and findings:**

Cross-sectional data trend analysis of 24.5 million adult medical claims was conducted using medical and pharmacy data (2012–2019) for adults aged 21–64 from the IBM Watson MarketScan Medicaid Multi-State Database. Information on sex, age, race, provider type, acute or chronic pain, and prescriptions for opioids and antidepressant and/or antianxiety medication from outpatient encounters were analyzed. For those receiving opioid prescriptions within 14 days of a pain diagnosis, ICD-10-CM codes were used to categorize diagnoses as chronic pain (back pain, neck pain, joint pain, and headache); or acute pain (dental-, ENT-, and orthopedic-related pain). Nearly 8 million adults had at least one prescription for antidepressant or antianxiety medications (MHD), with 2.5 million of those (32%) also diagnosed with an acute or chronic pain condition (pain + MHD). Among the pain + MHD group, 34% (0.85 million) received an opioid prescription within 14 days of diagnosis. Individuals with chronic pain diagnoses received a higher proportion of opioid prescriptions than those with acute pain. Among individuals with pain + MHD, the majority were aged 50–64 (35%), female (72%), and non-Hispanic white (65.1%). Nearly half (48.2%) of the opioid prescriptions given to adults with an MHD were provided by physicians. Compared to other physician types, Health Care Providers (HCPs) in emergency departments were 50% more likely to prescribe an opioid for dental pain to those with an MHD, whereas dentists were only half as likely to prescribe an opioid for dental pain management. Although overall opioid prescriptions for pain management declined from 2012 to 2019, adults with an MHD received opioids for pain management at nearly twice the level as adults without an MHD.

**Conclusions:**

Although HCPs have reduced opioids for acute or chronic pain to patients at high-risk for SUD, for example, those with MHD, the use of opioids for pain management has remained at consistently higher levels for this SUD high-risk group, suggesting the need to revisit pain management guidelines for those receiving antidepressant or antianxiety drugs.

## Introduction

Depression is the leading cause of disability in the United States (US) for those aged 15–44 [1]. Among adults 20 and older, about 8% experience depression in any given 2-week period [2]. Globally, it is estimated that depression affects 5% of adults and is also the leading cause of disability worldwide [3]. Major depression is a common, treatable mental health disorder (MHD) characterized by physical and cognitive symptoms, including changes in mood, disturbed sleep and appetite, and diminished interest in daily activities [4]. Anxiety disorders are the most common psychological illnesses in the US, affecting 19% of the population annually [5]. Anxiety disorders vary in the situations and objects to which they relate (e.g., social situations, specific phobias) but are marked by excessive anxiety and impairment in functioning [5].

Although depression and anxiety disorders have different etiologies that manifest their own unique emotional and behavioral symptoms, patients diagnosed with either condition often experience related symptoms like nervousness, irritability, and problems sleeping and concentrating [6]. Treatments for depression and anxiety are similar, initially started with psychotherapy and sometimes followed by prescription medications collectively classified as antidepressant/antianxiety drugs. Common examples of these drug classes include benzodiazepines, selective serotonin reuptake inhibitors (SSRIs), serotonin-norepinephrine reuptake inhibitors (SNRIs), tricyclic antidepressants, monoamine oxidase inhibitors, beta-blockers, and other anxiolytic and sedative/hypnotic drugs [7].

The relationship between pain and depressive symptoms is bi-directional [8]. Chronic pain is linked to depression, and patients diagnosed with depression often report systemic pain as psychological symptoms, often worsened by the pain experience [9, 10]. Pain and depression operate in a cycle by which depressive symptoms are exacerbated by pain, which is, in turn, worsened by symptoms of depression [11, 12] and even the memory of pain [13]. Sustained stress can also be an important influencer of chronic pain, creating a discordant cycle [14] which can further induce depression [15–17]. Consequently, it is estimated that as many as four out of five individuals with chronic pain also experience severe depression [18, 19].

Opioids are commonly used for the treatment of chronic pain. Because of the link between chronic pain and MHDs, nearly 50% of all opioid prescriptions are prescribed to patients with anxiety, depression, or other MHDs [20]. Although individuals with MHDs are at increased risk of misusing opioids [15], they are prescribed opioids at a higher rate than the general population [21]. It is estimated that 19% of the 38.6 million adults with MHDs use prescription opioids, compared to 5% of the general population [20].

In 2019, 9.5 million Americans aged 18 and older reported having a co-occurring MHD and substance use disorder (SUD) [22]. The concurrent use of opioids with antidepressant or antianxiety medication is of concern and requires careful consideration and vigilance due to the increased likelihood of developing a SUD, especially with long-term opioid use [23]. For example, individuals taking benzodiazepines (an antianxiety drug) are more likely to be prescribed opioids; in addition, they are at a higher risk of accidental overdose [24]. According to the National Institute on Drug Abuse, overdose deaths involving benzodiazepines rose more than four-fold from 1999 to 2017 (1,135 to 11,537), facilitated by the co-use of a benzodiazepine with an opioid [25, 26]. Previous research has suggested that people who are prescribed benzodiazepines and opioids are four to ten times more likely to overdose compared to those who are prescribed opioids alone [27]. Combining opioids and benzodiazepines can be unsafe because both drug types sedate users and suppress breathing—the leading cause of overdose fatalities [24].

Long-term use of opioids can increase both the risk of depression and sensitivity to pain, which in turn may also lead to depression [27, 28]. Compared to individuals taking opioids for 1–30 days, those taking opioids for 31–90 days had an 18% higher risk for depression [29]. Duration of opioid use by patients with a history of depression was found to be three times longer than patients without depression [30]. Although prescribing guidelines recommend against long-term opioid therapy for individuals with chronic pain and severe depression or anxiety, individuals with a history of major depression often are prescribed opioids for acute and chronic pain management [8].

Information pertaining to receipt of opioid drugs for pain-related conditions in patients among with an MHD is limited, especially within low-income populations such as Medicaid beneficiaries. This is important because low-income populations are prescribed opioid drugs at twice the rate of higher-income or non-Medicaid insured populations and are three to six times more likely to overdose [31]. The purpose of this study is to assess opioid prescribing patterns for acute or chronic pain management in patients pharmaceutically treated for depression or anxiety and to better understand whether prescribing guidelines are being followed.

## Methods

### Data source

Medicaid medical and pharmacy claims data from the IBM Watson MarketScan Research Medicaid Database were analysed [32]. These data cover 13 U.S. states and contain no geographic or personal identifiers. Person-level data (e.g., age, gender, and enrolment period) and claims-level data (e.g., outpatient pharmacy prescription claims) for the period January 1 2012 through December 31 2019, were extracted using Structured Query Language (SQL); records of all Medicaid patients who received ambulatory or outpatient health care were extracted to obtain relevant person-level data. Only data from adults ages 21–64 were used.

From these records and outpatient pharmacy prescription claims, we formed an analytical dataset comprised of patients who had been prescribed antidepressant or antianxiety medications (including the therapeutic classes consisting of anticonvulsants, benzodiazepines, and anxiolytic/sedative/hypnotics). The unit of analysis was defined as unique patient-year combinations. The same enrolment identification code could count as multiple patients with prescriptions for antidepressant or antianxiety medications if they received medications in more than one calendar year. The prescription date was defined as the date a patient first received an antidepressant or antianxiety medication each year.

Each patient’s chief complaint was categorized using the International Classification of Diseases, Ninth Revision, Clinical Modification diagnostic codes as one of the following: back pain, neck pain (cervicalgia), joint pain (osteoarthritis and rheumatoid arthritis), pain caused by orthopedic-related conditions (simple closed fractures, muscle strains or sprains), headache-related pain (including cluster headaches and migraine pain), pain caused by dental-related conditions (excluding temporomandibular joint conditions), or ear, nose, and throat (ENT)-related pain (otalgia). These seven conditions were further categorized to broadly represent various pain conditions classified as *chronic* (back pain, neck pain, joint pain, and headache) and *acute* (dental-, ENT-, and orthopedic-related pain) (shown in Appendix A, S1 Appendix). Patients were included if they had an outpatient claim with a diagnosis of one or more of the seven identified conditions within 90 days of an antidepressant or antianxiety medication prescription. To facilitate interpretation of results, if both acute and chronic conditions were listed on the same day, the acute condition was chosen. For the same reason, if more than one pain condition was listed for a patient, only the earliest-diagnosed condition was selected for inclusion.

Outpatient pharmacy claims were then searched for opioid-containing medications using the therapeutic class “opioid analgesics group.” This includes drugs derived from opium, including morphine, as well as semisynthetic and synthetic opioid agonists such as hydrocodone, oxycodone, and fentanyl. Individuals were selected who received a prescription for any opioid analgesic within 14 days of diagnosis for one of the seven specified pain-related conditions.

Records were then filtered to include only those patients enrolled for at least 14 continuous days in a Medicaid plan that included prescription drug coverage. The final analytical dataset was formed using medical and pharmacy records linked by the unique patient identifier corresponding to the visit timing previously described. Data used in this study was originally collected as part of administrative record processes and transformed into an analytical datafile containing no protected health information or other identifiers; thus, was exempted from review from the National Institutes of Health Institutional Review Board (NIH, IRB).

### Analytical variables

The receipt of an opioid prescription within 14 days of a pain diagnosis, categorized dichotomously (yes or no), was the primary outcome variable. Health care providers (HCPs) were categorized into emergency department providers (ED-HCPs), physicians, dentists, nurse practitioners or physician assistants (NP/PAs), and other HCPs. ‘Other HCPs’ included other all other provider types including unknown provider types. Patient variables included age group (21–29, 30–39, 40–49, 50–64), gender (male/female), and race/ethnicity (Hispanics, non-Hispanic white, African American, and other).

### Data analysis

Frequency distributions and proportions of patients with an opioid prescription were calculated by age group, gender, race/ethnicity, and provider type and then were stratified by pain condition type (chronic or acute). Individual multivariable logistic regression models were produced to ascertain the association of the key independent variable (pain conditions) with the dependent variable (receipt of an opioid within 14 days of diagnosis) while controlling for other covariates (age, HCPs, gender, and race/ethnicity). Analyses were performed using SAS software version 9.4 (SAS Institute Inc., Cary, NC, USA).

## Results

**Fig 1:**
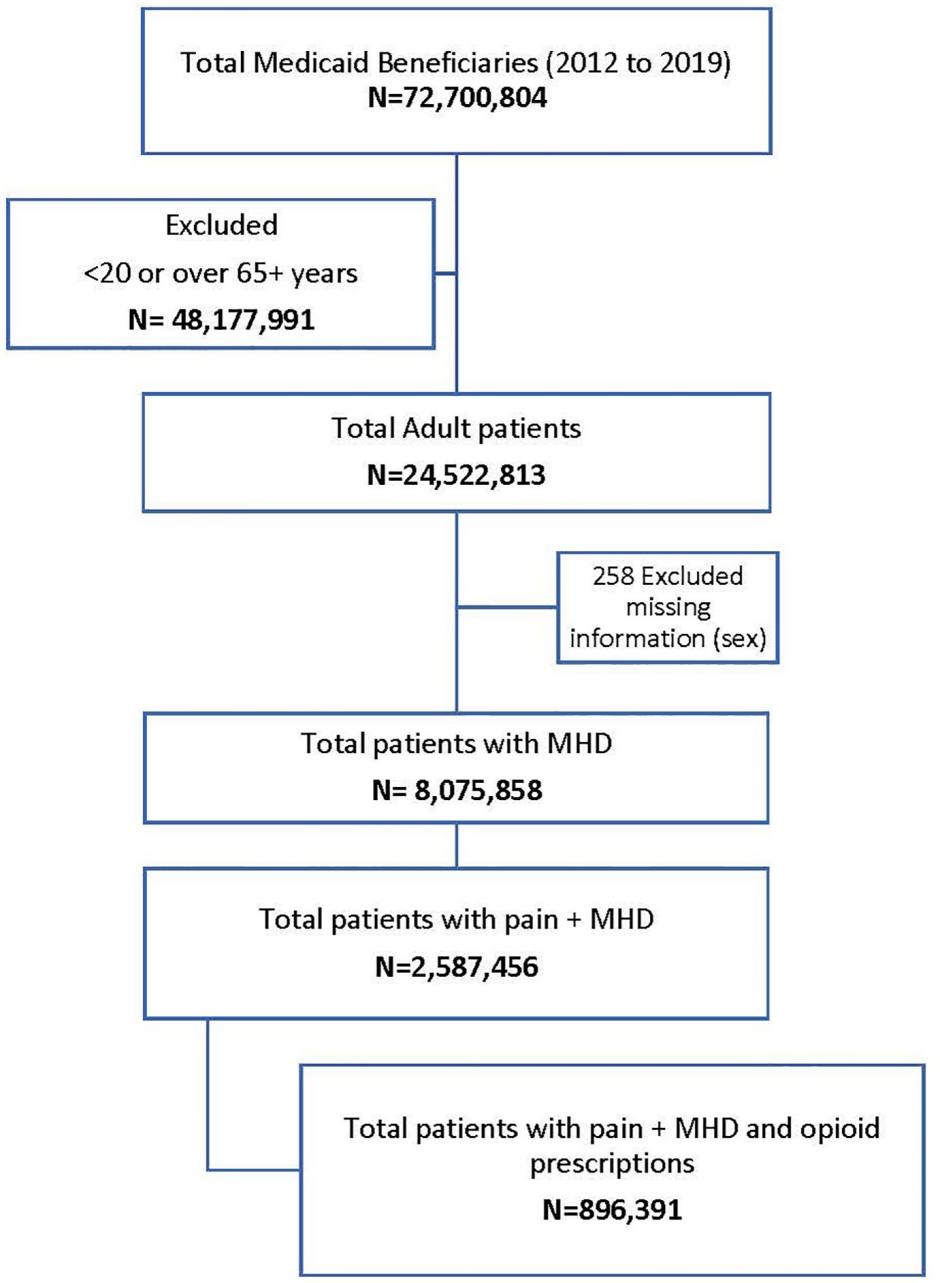
Cohort selection for the analytical sample of the study.

There were 72,700,804 Medicaid beneficiaries with relevant claims information during the study period. We excluded 48,177,991 individuals because they were either <20 years of age or 65 and older. From the remaining group, we excluded 258 persons because of missing information on gender and identified 8,075,858 patients receiving antidepressant or antianxiety medication (MHD). Among these individuals, 32.1% (2,587,456) had at least one of the seven pain conditions. Among these individuals receiving antidepressant or antianxiety medication and a pain diagnosis (pain + MHD), 34.6% (896,391) had an opioid prescription filled within 14 days (Fig 1).

**Fig 2:**
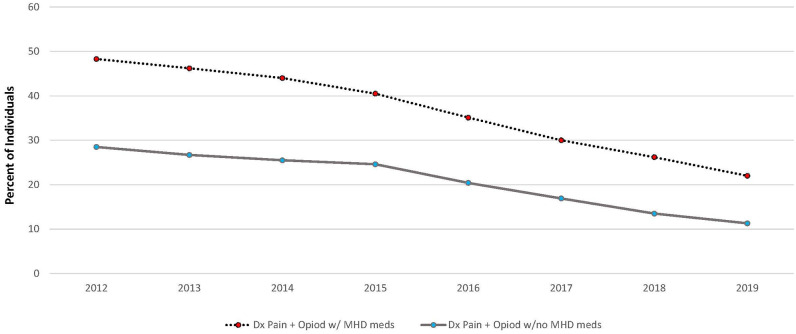
Proportion of individuals with a pain diagnosis receiving an opioid and either received anti-depressant or anxiety medications or have not been prescribed them. MHD meds: anti-Depression or anti-Anxiety Medication.

The proportion of persons with pain + MHD was generally stable from 2012 to 2015 (12.6%-13.6%). Opioid prescriptions increased in 2016 to 16.2% and gradually declined to 8.1% in 2019 (Table 1). During the study period, approximately 3 in 4 persons with pain + MHD received an opioid prescription for chronic pain. Overall, more than half of opioid prescriptions were for joint (27.6%) or back (27%) pain (chronic pain conditions), followed by orthopedic pain (18.1%). About 1 in 15 received an opioid prescription for dental pain (6.7%). The proportion of persons with pain and receiving an opioid was consistently higher for those also receiving anti-depressant or anxiety medications compared to those with no MHDs (Fig 2). Nearly half of those with a pain and receiving an opioid also received MHDs in 2012 and this declined to about 20% in 2019. Among those with a pain and receiving opioids but no MHDs, the prevalence declined from about 30% to 11% during the same period.

**Table 1:** Distribution of persons with anti-depression or antianxiety medications and pain by year.

Year	Medicaid Patients with Anti-Depression or Antianxiety Medications	Medicaid Patients with Anti-Depression or Antianxiety Medications and Pain Diagnosis	Medicaid Patients with Anti-Depression or Antianxiety Medications with Opioid Prescription															
			Acute Pain Conditions						Chronic Pain Conditions								Total	
			Dental		ENT		Orthopedics		Headache		Joint Pain		Back Pain		Neck Pain			
	n	n	n	%	n	%	n	%	n	%	n	%	n	%	n	%	n	%
**2012**	945,758	275,975	7,281	6.5	1,941	1.7	25,849	23.0	16,096	14.3	21,874	19.4	25,062	22.3	14,522	12.9	112,625	12.6
**2013**	885,017	253,410	7,105	6.3	1,901	1.7	24,686	22.0	15,490	13.8	22,832	20.4	24,703	22.0	15,376	13.7	112,093	12.5
**2014**	1,008,111	286,284	7,950	6.5	1,798	1.5	27,149	22.4	15,776	13.0	25,000	20.6	26,679	22.0	17,036	14.0	121,388	13.5
**2015**	1,115,144	316,596	8,180	6.7	1,797	1.5	25,928	21.3	14,223	11.7	27,508	22.6	28,351	23.3	15,622	12.8	121,609	13.6
**2016**	1,111,793	414,503	10,642	7.3	1,340	0.9	19,993	13.8	13,416	9.3	46,335	32.0	49,523	34.2	3,662	2.5	144,911	16.2
**2017**	1,114,295	399,232	8,813	7.4	1,118	0.9	16,259	13.6	10,812	9.0	40,988	34.2	38,844	32.4	3,048	2.5	119,882	13.4
**2018**	962,131	332,592	5,504	6.0	795	0.9	12,072	13.2	7,919	8.7	34,516	37.7	28,437	31.1	2,294	2.5	91,537	10.2
**2019**	933,609	308,864	4,497	6.2	645	0.9	10,150	14.0	6,148	8.5	28,277	39.1	20,764	28.7	1,865	2.6	72,346	08.1
**Total**	8,075,858	2,587,456	59,972	6.7	11,335	1.3	162,086	18.1	99,880	11.1	247,330	27.6	242,363	27.0	36,355	8.2	896,391	100

Among those aged 21–30, 42% received an opioid prescription for acute pain (dental, ENT, and orthopedic), whereas 41% received an opioid prescription for headache and back pain (Table 2). The proportion of opioid prescriptions for acute pain declined with age but substantially increased for one specific chronic pain diagnosis–joint pain, increasing from 9.7% for those aged 21–30 to 40.9% for those aged 51–64. Overall, more adults aged 51–64 received opioids for pain management compared to younger adults aged 21–30 (35% versus 14.3%). There was no obvious gender difference regarding opioid prescriptions for each of the seven pain conditions assessed. However, 72% of individuals with pain + MHD were female. Hispanics, Non-Hispanic whites, and African Americans received nearly similar proportions of opioid prescriptions for each of the seven pain conditions except for joint pain, which was higher for African Americans. Overall, 65.1% of opioid prescriptions were provided to non-Hispanic whites compared to 20.1% of African Americans and 1.4% of Hispanics.

**Table 2:** Demographic characteristics of persons with anti-depression or antianxiety medications and receiving opioid prescription for pain.

	Acute Pain Conditions						Chronic Pain Conditions									
Diagnoses	Dental		ENT		Orthopedic		Headache		Joint Pain		Back Pain		Neck Pain		Total	
Variables	n	%	n	%	n	%	n	%	n	%	n	%	n	%	N	%
**Total**	59,972	6.7%	11,335	1.3%	162,086	18.1%	99,880	11.1%	247,330	27.6%	242,363	27.0%	73,425	8.2%	896,391	100
**Age group in years**																
**21–30**	19,250	15.0%	2,661	2.1%	31,892	24.9%	21,300	16.6%	12,445	9.7%	31,768	24.8%	8,788	6.9%	128,104	14.3
**31–40**	19,526	8.7%	3,500	1.6%	48,716	21.6%	31,049	13.8%	39,985	17.7%	62,866	27.9%	19,930	8.8%	225,572	25.2
**41–50**	11,259	4.9%	2,582	1.1%	39,301	17.1%	24,423	10.7%	66,798	29.1%	63,530	27.7%	21,426	9.3%	229,319	25.6
**51–64**	9,937	3.2%	2,592	0.8%	42,177	13.5%	23,108	7.4%	128,102	40.9%	84,199	26.9%	23,281	7.4%	313,396	35.0
**Gender**																
**Male**	15,487	6.2%	1,857	0.7%	48,380	19.2%	18,100	7.2%	73,005	29.0%	73,446	29.2%	21,115	8.4%	251,390	28.0
**Female**	44,485	6.9%	9,478	1.5%	113,706	17.6%	81,780	12.7%	174,325	27.0%	168,917	26.2%	52,310	8.1%	645,001	72.0
**Race/ethnicity**																
**Non-Hispanic White**	40,776	7.0%	7,817	1.3%	108,632	18.6%	64,285	11.0%	150,010	25.7%	161,418	27.7%	50,547	8.7%	583,485	65.1
**African American**	10,883	6.0%	1,932	1.1%	28,847	16.0%	21,151	11.7%	59,308	32.9%	46,014	25.5%	12,092	6.7%	180,227	20.1
**Hispanic**	1,017	8.3%	174	1.4%	2,224	18.2%	1,540	12.6%	3,022	24.8%	3,229	26.5%	986	8.1%	12,192	1.4
**Other**	7,296	6.1%	1,412	1.2%	22,383	18.6%	12,904	10.7%	34,990	29.0%	31,702	26.3%	9,800	8.1%	120,487	13.4

Emergency department HCPs, NP/PAs, and dentists were the lowest prescribers of opioids, providing prescriptions to about 6% of the sample (Table 3). Among persons with an MHD also prescribed an opioid by ED-HCPs, about 40% did so for acute pain such as orthopedic (20.7%) and dental (19.7%) pain. Another 40% received an opioid for joint (24.3%) and back (16.2%) pain. In general, 62.5% of individuals receiving an opioid from other healthcare providers did so for joint and back pain. The exception was dentists, where at least half of people receiving an opioid did so for diagnoses associated with acute pain. Interestingly, among persons receiving an opioid from a dentist, about 28% did so for joint (14.4%) and back (14%) pain. Almost two-thirds of persons receiving an opioid for chronic pain management did so from multiple provider types (>70%).

**Table 3:** Age, gender, race/ethnicity, and pain diagnosis of persons with anti-depression or antianxiety medications and receiving opioid prescription for pain by healthcare provider types.

Health care providers	ED-HCPs		Physicians		NP/PA		Dentists		Others		Multiple Provider Types		Total	
	n	%^a^	n	%^a^	n	%^a^	n	%^a^	n	%^a^	n	%	N	%
**Total**	20,352	2.3	157,116	17.5	19,364	2.2	13,386	1.5	110,742	12.3	575,431	64.2	896,391	100%
**Age group in years**														
21–30	4,894	24.0%	20,043	12.8%	2,169	11.2%	4,179	31.2%	11,305	10.2%	85,514	14.9%	128,104	14.3%
31–40	6,201	30.5%	41,813	26.6%	5,204	26.9%	4,325	32.3%	23,566	21.3%	144,463	25.1%	225,572	25.2%
41–50	4,543	22.3%	41,891	26.7%	5,139	26.5%	2,670	19.9%	29,072	26.3%	146,004	25.4%	229,319	25.6%
51–64	4,714	23.2%	53,369	34.0%	6,852	35.4%	2,212	16.5%	46,799	42.3%	199,450	34.7%	313,396	35.0%
**Gender**														
Male	5,273	25.9%	45,258	28.8%	5,441	28.1%	3,119	23.3%	33,853	30.6%	158,446	27.5%	251,390	28.0%
Female	15,079	74.1%	111,858	71.2%	13,923	71.9%	10,267	76.7%	76,889	69.4%	416,985	72.5%	645,001	72.0%
**Race/ethnicity**														
Non-Hispanic white	11,073	54.4%	103,054	65.6%	12,708	65.6%	8,618	64.4%	72,726	65.7%	375,306	65.2%	583,485	65.1%
African American	3,633	17.9%	30,442	19.4%	3,906	20.2%	2,618	19.6%	21,965	19.8%	117,663	20.4%	180,227	20.1%
Hispanic	326	1.6%	2,171	1.4%	275	1.4%	364	2.7%	1,143	1.0%	7,913	1.4%	12,192	1.4%
Others	5,320	26.1%	21,449	13.7%	2,475	12.8%	1,786	13.3%	14,908	13.5%	74,549	13.0%	120,487	13.4%
**Pain Diagnosis**														
Dental	4,018	19.7%	4,950	3.2%	828	4.3%	5,529	41.3%	4,372	3.9%	40,275	7.0%	59,972	6.7%
ENT	519	2.6%	1,769	1.1%	229	1.2%	255	1.9%	880	0.8%	7,683	1.3%	11,335	1.3%
Orthopedic	4,210	20.7%	22,656	14.4%	1,850	9.6%	1,226	9.2%	15,285	13.8%	116,859	20.3%	162,086	18.1%
Headache	2,196	10.8%	17,960	11.4%	1,716	8.9%	1,956	14.6%	10,272	9.3%	65,780	11.4%	99,880	11.1%
Back Pain	3,298	16.2%	46,197	29.4%	5,971	30.8%	1,874	14.0%	37,052	33.5%	152,938	26.6%	247,330	27.6%
Joint Pain	4,940	24.3%	49,435	31.5%	6,675	34.5%	1,926	14.4%	32,205	29.1%	147,182	25.6%	242,363	27.0%
Neck Pain	1,171	5.8%	14,149	9.0%	2,095	10.8%	620	4.6%	10,676	9.6%	44,714	7.8%	73,425	8.2%

^a^ Those who saw more than 1 provider type were excluded from this analysis.

Table 4 presents the multivariable modeling results indicating the likelihood of receiving an opioid for a particular pain diagnosis. Older age groups (31–40, 41–50, and 51–64) were consistently more likely to receive an opioid prescription compared to those aged 21–30 for all pain conditions except dental. Females were less likely to receive an opioid for back, joint, and neck pain compared to males but were more likely to receive an opioid for orthopedic and dental pain than males. Dentists were less than half as likely (OR 0.44 (95% CI = 0.42–0.46)) to prescribe opioids for dental pain compared to physicians; however, ED-HCPs were 50% more likely to prescribe opioids for dental pain (OR 1.54 (95% CI = 1.50–1.58)) compared to physicians when controlling for patient characteristics such as age, gender, and race/ethnicity. Compared to physicians, ED-HCPs were less likely to prescribe opioids for chronic back pain (OR 0.69 (95% CI = 0.68–0.70)), joint pain (OR 0.80 (95% CI = 0.79–0.81)), and neck pain (OR, 0.69 (95% CI = 0.68–0.71)), whereas NP/PAs were more likely to prescribe opioids for back pain (OR 1.05 (95% CI = 1.03–1.08)), joint pain (OR 1.02 (95% CI = 1.00–1.05)), and neck pain (OR, 1.25 (95% CI = 1.20–1.31)). In general, other provider types were less likely to prescribe opioids for any of the seven pain conditions compared to physicians.

**Table 4:** Multivariable regression results estimating the probability of receiving an opioid for a pain diagnosis.

	Acute Pain Conditions			Chronic Pain Conditions			
Pain Diagnosis	Dental	ENT	Orthopedic	Headache	Back Pain	Joint Pain	Neck Pain
N	1,61,445	52,370	4,13,792	4,47,405	7,35,349	6,00,349	1,76,746
% Patients with opioid prescriptions	37.1%	21.6%	39.2%	22.3%	33.6%	40.4%	41.5%
**Age group, years (Reference = 21–30)**							
31–40	1.00 (0.98–1.03)	1.29 (1.22–1.36)	1.31 (1.28–1.33)	1.29 (1.27–1.32)	1.56 (1.52–1.6)	1.40 (1.38–1.42)	1.29 (1.25–1.33)
41–50	0.87 (0.84–0.89)	1.39 (1.31–1.48)	1.30 (1.28–1.33)	1.43 (1.4–1.46)	1.72 (1.68–1.76)	1.47 (1.45–1.49)	1.28 (1.24–1.33)
51–64	0.72 (0.69–0.74)	1.37 (1.29–1.46)	1.14 (1.12–1.16)	1.5 (1.47–1.54)	1.67 (1.64–1.71)	1.48 (1.46–1.51)	1.26 (1.22–1.30)
**Gender (Reference = Male)**							
Female	1.34 (1.31–1.37)	1.03 (0.98–1.09)	1.07 (1.05–1.08)	1.07 (1.05–1.09)	0.94 (0.93–0.95)	0.98 (0.97–0.99)	0.91 (0.89–0.93)
**Race (Reference = non-Hispanic white)**							
African American	1.04 (1.01–1.06)	0.89 (0.84–0.94)	0.98 (0.96–0.99)	0.88 (0.86–0.89)	1.00 (0.99–1.01)	0.91 (0.9–0.93)	0.97 (0.94–0.99)
Hispanic	0.90 (0.83–0.97)	0.78 (0.66–0.93)	0.88 (0.84–0.93)	0.70 (0.66–0.74)	0.76 (0.73–0.79)	0.74 (0.71–0.77)	0.75 (0.70–0.82)
Other	0.94 (0.91–0.97)	0.86 (0.81–0.92)	0.96 (0.95–0.98)	0.87 (0.85–0.89)	1.00 (0.99–1.02)	0.92 (0.91–0.94)	0.93 (0.90–0.96)
**Healthcare Providers Type (Reference = Physician)**							
Dentist	0.44 (0.42–0.46)						
ED HCPS	1.54 (1.50–1.58)	1.44 (1.37–1.52)	1.01 (1.00–1.03)	1.00 (0.98–1.02)	0.69 (0.68–0.7)	0.80 (0.79–0.81)	0.69 (0.68–0.71)
NP & PA	1.04 (0.99–1.09)	0.89 (0.82–0.97)	0.87 (0.84–0.90)	0.85 (0.82–0.88)	1.05 (1.03–1.08)	1.02 (1.00–1.05)	1.25 (1.20–1.31)
Others	0.87 (0.85–0.90)	0.97 (0.92–1.03)	0.85 (0.84–0.87)	0.90 (0.89–0.92)	0.82 (0.81–0.83)	0.82 (0.81–0.83)	0.70 (0.69–0.72)
Goodness of Fit.	101.9 DF = 8 < .0001	55.9 DF = 8 .0002	271.9 DF = 8 < .0001	107.0 DF = 8 < .0001	275.5 DF = 8 < .0001	299.0 DF = 8 < .0001	66.6 DF = 8 .0003
% of Cases Correctly Classified (prob level = 0.32)	52.0%	78.4%	39.5%	77.7%	45.5%	43.5%	42.2%

## Discussion

The opioid crisis continues in the U.S., as evidenced by the fact that opioids accounted for nearly 50,000 overdose deaths in 2019, which is more than 70% of all drug overdose deaths [33]. Approximately one in four people receiving opioids for pain management misuse them, and about one in 10 people using an opioid for chronic pain develop an SUD [34]. Although most of the global population who are dependent on opioids use heroin, the proportion of those using prescription opioids is also increasing, raising concerns about the over-prescription of opioids [21, 35]. The relationships between SUD, depression, and anxiety are most likely bi-directional [36]. Findings from our study suggest that among adults with MHD, approximately 35% had an opioid prescription filled and nearly three in four were prescribed opioids to manage chronic pain.

The proportion of Medicaid enrollees with pain + MHD was generally stable from 2012 to 2015, increased in 2016, but then gradually declined until 2019. This trend parallels the overall national opioid dispensing rate, which declined from 2012 to 2019, falling to its lowest rate in 14 years, at 46.7 prescriptions per 100 persons (a total of more than 153 million opioid prescriptions) [33]. This peak coincides with a declaration from the National Health Emergency of the opioid crisis in October 2017. However, prescription opioids were still involved in over 28% of all opioid overdose deaths in 2019, a nearly 7% decrease in prescription opioid-involved death rates from the prior year.

Findings from our study show that for Medicaid beneficiaries with an MHD, the highest percentage of opioid prescriptions for acute pain conditions was for orthopedic pain (18%), followed by dental pain (6.7%). Joint (28%) and back (27%) pain resulted in opioid prescriptions most frequently for chronic pain conditions. However, some studies of musculoskeletal injuries (orthopedic pain) have found no association between pain intensity and severity of the injury, supporting the observation that pain perception is multifactorial [37–39]. Variations in pain intensity and magnitude of limitations are affected by psychosocial aspects of illness in addition to measures of pathophysiology, which may lead to increased risk for an opioid overdose for patients with an existing MHD [40]. Pain from dental-related conditions is generally acute in nature, and emergency department visits for pain relief are commonplace in the U.S., especially among lower-income adults [41]. Opioid prescriptions, even for a short duration, can lead to long-term use. The likelihood of chronic use begins to increase after the third day, rising rapidly thereafter [42]. It has also been observed that opioid use after third molar extraction may be a young person’s first experience with opioids and may lead to chronic opioid use [43].

For chronic pain such as back and joint pain which affects individual daily functioning and well-being, women, older age groups, socioeconomic status, and race or ethnicity may lead to coping strategies that progress to more depressive symptoms [44]. Prescribing opioid medications to patients with a history of depression may have a synergistic effect that results in overdose or misuse of medications.

Notably, the increase in opioid prescriptions over time was not associated with parallel increases in nonopioid pain relievers or the proportion of ambulatory pain patients receiving pharmacologic treatment [45]. Without improvement in patient outcomes, these prescriptions are needlessly provided to patients and are associated with an increased risk of abuse. Overprescribing also leads to unused opioids, creating the possibility of nonmedical usage or diversion [46]. Furthermore, for patients who receive a first opioid prescription of any duration, 21% progress to receiving more prescriptions episodically, and 6% progress to long-term use [47]. Up to half of the patients who take opioids for at least three months remain on opioids five years later and are likely to become lifelong users [27, 48, 49]. This is an important finding because current pain management guidelines recommend an opioid prescription as the last option to manage pain in patients with a history of depression [23, 25, 26].

Depression is a risk factor for opioid overdose [15, 26, 50–54]. Previous studies suggest the relationship between MHDs like depression or anxiety and patterns of opioid use in these patients is complex [9, 15, 50, 53, 55]. Pain is commonly associated with MHDs and acts as the moderator in the relationship between pain and opioid use. This relationship is concerning as depression is also a prominent risk factor for opioid misuse, overdose, and other adverse outcomes. Studies have shown that the use of opioid analgesics in patients even without depression at the time of opioid initiation may increase the risk of developing Major Depressive Disorder [52, 53]. A temporal association has been demonstrated in these relationships, which supports the hypothesis that prescription opioid analgesics may be a causal contributor to depression [53]. This causal effect may increase when the patient who is prescribed opioids for pain treatment has a preexisting depression.

Because depression severity is an important factor contributing to misuse of prescribed opioids among chronic pain patients [56], appropriate screening and monitoring are important considerations for managing risks. Our study found variability in prescription rates among various health care providers, with physicians and ED-HCPs more likely to prescribe opioids to patients with acute dental pain, orthopedic pain, and ENT pain, and NP/PAs more likely to prescribe opioids for neck pain but less likely to prescribe opioids for ENT, orthopedic, and headache pain. Dentists were less likely to prescribe opioids for dental pain compared to all other providers, presumably because dentists have the necessary skills and equipment to provide definitive rather than palliative treatment for dental problems. Further, the American Dental Association (along with the Centers for Disease Control and Prevention and several states) encourage dentists to limit the amount and duration of opioids prescribed [57, 58]. Older age groups (>30) were consistently more likely to receive opioid prescriptions compared to the 21–29 age group for all pain conditions except dental. This age-related increase parallels the use of antidepressant or antianxiety medications, pointing towards the risk of opioid overuse, abuse, or overdose in these patients. Compared to men, women were twice as likely to get opioid prescriptions for all pain conditions except neck and joint pain. While men are more likely to die from prescription overdose than women [59], overdoses related to opioids have greatly increased in women compared to men [59]. Additionally, pain sensitivity varies between men and women, with women reporting more chronic conditions that cause pain [60]; these conditions may be associated with higher opioid prescription rates.

This study makes it clear that many patients receiving pharmacotherapy for depression or anxiety were also receiving opioid prescriptions for pain, in spite of guidelines warning against this practice. The concern arises whether some healthcare providers may be ignorant of clinical recommendations, may not have an accurate picture of the patient’s health care history, or may overlook relevant medical information when prescribing these medications. Eliciting a history of antidepressant medication is often communicated by patients more readily than a diagnosis of depression or anxiety [61]. Despite current evidence-based opioid prescribing guidelines [23, 25, 26], other factors such as unconscious bias or conflict avoidance may contribute to a healthcare provider’s decision whether to prescribe an opioid [29, 62].

The danger of co-prescribing opioids and antianxiety drugs such as benzodiazepines is well established. For example, in a 2015 study, 23 percent of people who died of an opioid overdose also tested positive for benzodiazepines [63]. In North Carolina, the overdose death rate for individuals taking both opioids and benzodiazepines together was ten times higher than among those receiving opioids alone [18], while in Canada, 60% of individuals who died from an opioid overdose tested positive for benzodiazepines [64]. Among U.S. veterans, co-prescription of opioid and benzodiazepine drugs was associated with an increased risk of drug overdose death [65].

A recent review of systematic reviews has raised important concerns regarding the effectiveness of antidepressants when prescribing for chronic pain management and an accompanying commentary suggested that clinical practice guidelines should be revisited as a result of this study’s findings [66, 67]. Our findings suggest that continuing education focusing on prescribing guidelines is warranted for health care providers when treating pain conditions, especially in acute and emergency settings. Equally important, interprofessional continuing education focusing on prescribing guidelines for pain among patients with an MHD would be beneficial. Likewise, clinical decision support alerts triggered by opioid prescriptions that promote checking MHD history and pharmacy dispensation data (including drug-problem lists and drug interaction alerts), if implemented correctly, can improve guideline adherence [68].

Because our study was limited to a Medicaid cohort, our findings are not generalizable to the U.S. population. Additionally, the study design prevents us from drawing any conclusions pertaining to causality, and diagnostic misclassification is possible, but this would be considered a non-systematic bias. Nevertheless, the claims data obtained for our analyses originate from a large sample comprising a significant percentage of the Medicaid beneficiaries in the U.S. and provides real-world population exploratory findings. Our study used opioid prescription as the unit of opioid exposure. Because the number and potency of the prescribed drugs vary considerably, this may more or less be an accurate proxy for opioid dosage. Appendix B (S1 Appendix) shows the distribution of morphine milligram equivalents (MME) for pain conditions by anti-depression/anxiety medications, pain type, and age group. Using MME as a more accurate measure of opioid exposure may provide additional insight and refinement for future studies.

## Conclusion

Although healthcare providers have reduced opioid prescriptions for acute or chronic pain for patients at high risk for SUD (including those with MHDs), opioid use for pain management has remained at consistently high levels for this high-risk group. This suggests a need to revisit and amplify pain management guidelines for providers with patients receiving anti-depression and antianxiety drugs.

## Supporting information

S1 Appendix (DOCX)

## References

[1] CDC. Mental Health | CDC. Cent. Dis. Control Prev. 2022. https://www.cdc.gov/mentalhealth/index.htm (accessed 19 Apr 2022).

[2] Products—Data Briefs—Number 303—February 2018. 2019. https://www.cdc.gov/nchs/products/databriefs/db303.htm (accessed 19 Apr 2022).

[3] Depression. https://www.who.int/news-room/fact-sheets/detail/depression (accessed 19 Apr 2022).

[4] Major depressive disorder—PubMed. https://pubmed.ncbi.nlm.nih.gov/27629598/ (accessed 19 Apr 2022).

[5] Any Anxiety Disorder. Natl. Inst. Ment. Health NIMH. https://www.nimh.nih.gov/health/statistics/any-anxiety-disorder (accessed 19 Apr 2022).

[6] NIMH » Home. https://www.nimh.nih.gov/ (accessed 19 Apr 2022).

[7] Strawn JR, Geracioti L, Rajdev N, et al. Pharmacotherapy for Generalized Anxiety Disorder in Adults and Pediatric Patients: An Evidence-Based Treatment Review. *Expert Opin Pharmacother* 2018;19:1057–70. doi: 10.1080/14656566.2018.1491966 30056792 PMC6340395

[8] Lerman SF, Rudich Z, Brill S, et al. Longitudinal associations between depression, anxiety, pain, and pain-related disability in chronic pain patients. *Psychosom Med* 2015;77:333–41. doi: 10.1097/PSY.0000000000000158 25849129

[9] Fishbain DA, Cutler R, Rosomoff HL, et al. Chronic pain-associated depression: antecedent or consequence of chronic pain? A review. *Clin J Pain* 1997;13:116–37. doi: 10.1097/00002508-199706000-00006 9186019

[10] Hermesdorf M, Berger K, Baune BT, et al. Pain Sensitivity in Patients With Major Depression: Differential Effect of Pain Sensitivity Measures, Somatic Cofactors, and Disease Characteristics. *J Pain* 2016;17:606–16. doi: 10.1016/j.jpain.2016.01.474 26867484

[11] Agüera-Ortiz L, Failde I, Mico JA, et al. Pain as a symptom of depression: prevalence and clinical correlates in patients attending psychiatric clinics. *J Affect Disord* 2011;130:106–12. doi: 10.1016/j.jad.2010.10.022 21055826

[12] Woo AK. Depression and Anxiety in Pain. *Rev Pain* 2010;4:8–12. doi: 10.1177/204946371000400103 26527193 PMC4590059

[13] Frequency of painful physical symptoms with major depressive disorder in asia: relationship with disease severity and quality of life—PubMed. https://pubmed.ncbi.nlm.nih.gov/19192462/ (accessed 19 Apr 2022). 10.4088/jcp.08m04114 19192462

[14] von Knorring L, Perris C, Eisemann M, et al. Pain as a symptom in depressive disorders. II. Relationship to personality traits as assessed by means of KSP. *Pain* 1983;17:377–84. doi: 10.1016/0304-3959(83)90169-0 6664683

[15] Davis MA, Lin LA, Liu H, et al. Prescription Opioid Use among Adults with Mental Health Disorders in the United States. *J Am Board Fam Med JABFM* 2017;30:407–17. doi: 10.3122/jabfm.2017.04.170112 28720623

[16] Ehrich E, Turncliff R, Du Y, et al. Evaluation of opioid modulation in major depressive disorder. *Neuropsychopharmacol Off Publ Am Coll Neuropsychopharmacol* 2015;40:1448–55. doi: 10.1038/npp.2014.330 25518754 PMC4397403

[17] Howe CQ, Sullivan MD. The missing ‘P’ in pain management: how the current opioid epidemic highlights the need for psychiatric services in chronic pain care. *Gen Hosp Psychiatry* 2014;36:99–104. doi: 10.1016/j.genhosppsych.2013.10.003 24211157

[18] Dasgupta N, Funk MJ, Proescholdbell S, et al. Cohort Study of the Impact of High-Dose Opioid Analgesics on Overdose Mortality. *Pain Med Malden Mass* 2016;17:85–98. doi: 10.1111/pme.12907 26333030

[19] Abuse NI on D. Overdose Death Rates. Natl. Inst. Drug Abuse. 2022. https://nida.nih.gov/drug-topics/trends-statistics/overdose-death-rates (accessed 19 Apr 2022).

[20] Salas J, Scherrer JF, Schneider FD, et al. New-onset depression following stable, slow, and rapid rate of prescription opioid dose escalation. *Pain* 2017;158:306–12. doi: 10.1097/j.pain.0000000000000763 28092649 PMC7050294

[21] Crofford LJ. Adverse effects of chronic opioid therapy for chronic musculoskeletal pain. *Nat Rev Rheumatol* 2010;6:191–7. doi: 10.1038/nrrheum.2010.24 20357788

[22] Home | SAMHDA. https://www.datafiles.samhsa.gov/ (accessed 19 Apr 2022).

[23] Korownyk CS, Montgomery L, Young J, et al. PEER simplified chronic pain guideline: Management of chronic low back, osteoarthritic, and neuropathic pain in primary care. *Can Fam Physician Med Fam Can* 2022;68:179–90. doi: 10.46747/cfp.6803179 35292455 PMC9833192

[24] Abuse NI on D. Benzodiazepines and Opioids. Natl. Inst. Drug Abuse. 2021. https://nida.nih.gov/drug-topics/opioids/benzodiazepines-opioids (accessed 19 Apr 2022).

[25] Manchikanti L, Kaye AM, Knezevic NN, et al. Responsible, Safe, and Effective Prescription of Opioids for Chronic Non-Cancer Pain: American Society of Interventional Pain Physicians (ASIPP) Guidelines. *Pain Physician* 2017;20:S3–92. 28226332

[26] Noninvasive Treatments for Acute, Subacute, and Chronic Low Back Pain: A Clinical Practice Guideline From the American College of Physicians—PubMed. https://pubmed.ncbi.nlm.nih.gov/28192789/ (accessed 18 Apr 2022). 10.7326/M16-2367 28192789

[27] Braden JB, Sullivan MD, Ray GT, et al. Trends in long-term opioid therapy for noncancer pain among persons with a history of depression. *Gen Hosp Psychiatry* 2009;31:564–70. doi: 10.1016/j.genhosppsych.2009.07.003 19892215 PMC2774904

[28] Traylor C. Medicaid Strategies for Non-Opioid Pharmacologic and Non-Pharmacologic Chronic Pain Management.;: 14.

[29] Chou R, Fanciullo GJ, Fine PG, et al. Clinical guidelines for the use of chronic opioid therapy in chronic noncancer pain. *J Pain* 2009;10:113–30. doi: 10.1016/j.jpain.2008.10.008 19187889 PMC4043401

[30] Pozzi A, Gallelli L. Pain management for dentists: the role of ibuprofen. *Ann Stomatol (Roma)* 2012;2:3–24. PMC3414241 22888399

[31] Becker DE. Pain management: Part 1: Managing acute and postoperative dental pain. *Anesth Prog* 2010;57:67–78; quiz 79–80. doi: 10.2344/0003-3006-57.2.67 20553137 PMC2886920

[32] MarketScan Research Databases—Databases. 2022. https://www.ibm.com/products/marketscan-research-databases/databases (accessed 19 Apr 2022).

[33] Drug Overdose Deaths in the U.S. Top 100,000 Annually. 2021. https://www.cdc.gov/nchs/pressroom/nchs_press_releases/2021/20211117.htm (accessed 19 Apr 2022).

[34] Abuse NI on D. Opioid Overdose Crisis. Natl. Inst. Drug Abuse. 2021.https://nida.nih.gov/drug-topics/opioids/opioid-overdose-crisis (accessed 19 Apr 2022).

[35] Opioid overdose. https://www.who.int/news-room/fact-sheets/detail/opioid-overdose (accessed 19 Apr 2022).

[36] Rogers AH, Zvolensky MJ, Ditre JW, et al. Association of opioid misuse with anxiety and depression: A systematic review of the literature. *Clin Psychol Rev* 2021;84:101978. doi: 10.1016/j.cpr.2021.101978 33515811

[37] Bot AGJ, Bekkers S, Arnstein PM, et al. Opioid use after fracture surgery correlates with pain intensity and satisfaction with pain relief. *Clin Orthop* 2014;472:2542–9. doi: 10.1007/s11999-014-3660-4 24777731 PMC4079891

[38] Briet JP, Houwert RM, Hageman MGJS, et al. Factors associated with pain intensity and physical limitations after lateral ankle sprains. *Injury* 2016;47:2565–9. doi: 10.1016/j.injury.2016.09.016 27659849

[39] Kadzielski JJ, Bot AGJ, Ring D. The influence of job satisfaction, burnout, pain, and worker’s compensation status on disability after finger injuries. *J Hand Surg* 2012;37:1812–9. doi: 10.1016/j.jhsa.2012.05.023 22763059

[40] Preuss CV, Kalava A, King KC. Prescription of Controlled Substances: Benefits and Risks. In: *StatPearls*. Treasure Island (FL):: StatPearls Publishing 2022. http://www.ncbi.nlm.nih.gov/books/NBK537318/ (accessed 19 Apr 2022). 30726003

[41] Cohen LA, Harris SL, Bonito AJ, et al. Low-Income and Minority Patient Satisfaction with Visits to Emergency Departments and Physician Offices for Dental Problems. *J Am Coll Dent* 2009;76:23. 19928365 PMC2819232

[42] Shah A, Hayes CJ, Martin BC. Characteristics of Initial Prescription Episodes and Likelihood of Long-Term Opioid Use—United States, 2006–2015. *MMWR Morb Mortal Wkly Rep* 2017;66:265–9. doi: 10.15585/mmwr.mm6610a1 28301454 PMC5657867

[43] Persistent Opioid Use After Wisdom Tooth Extraction | Addiction Medicine | JAMA | JAMA Network. https://jamanetwork.com/journals/jama/fullarticle/2695661 (accessed 19 Apr 2022).

[44] Classification of Chronic Pain, Second Edition (Revised). Int. Assoc. Study Pain IASP. https://www.iasp-pain.org/publications/free-ebooks/classification-of-chronic-pain-second-edition-revised/ (accessed 19 Apr 2022).

[45] Ambulatory diagnosis and treatment of nonmalignant pain in the United States, 2000–2010—PubMed. https://pubmed.ncbi.nlm.nih.gov/24025657/ (accessed 19 Apr 2022). 10.1097/MLR.0b013e3182a95d86 PMC3845222 24025657

[46] Inciardi JA, Surratt HL, Lugo Y, et al. The Diversion of Prescription Opioid Analgesics. *Law Enforc Exec Forum* 2007;7:127–41. 25267926 PMC4176900

[47] Hooten WM, St Sauver JL, McGree ME, et al. Incidence and Risk Factors for Progression From Short-term to Episodic or Long-term Opioid Prescribing: A Population-Based Study. *Mayo Clin Proc* 2015;90:850–6. doi: 10.1016/j.mayocp.2015.04.012 26141327 PMC4548808

[48] Long-term chronic opioid therapy discontinuation rates from the TROUP study—PubMed. https://pubmed.ncbi.nlm.nih.gov/21751058/ (accessed 19 Apr 2022). 10.1007/s11606-011-1771-0 PMC3235603 21751058

[49] Von Korff M, Korff MV, Saunders K, et al. De facto long-term opioid therapy for noncancer pain. *Clin J Pain* 2008;24:521–7. doi: 10.1097/AJP.0b013e318169d03b 18574361 PMC3286630

[50] Sullivan MD. Depression Effects on Long-term Prescription Opioid Use, Abuse, and Addiction. *Clin J Pain* 2018;34:878–84. doi: 10.1097/AJP.0000000000000603 29505419

[51] Kroenke K, Wu J, Bair MJ, et al. Reciprocal Relationship between Pain and Depression: A 12-Month Longitudinal Analysis in Primary Care. *J Pain Off J Am Pain Soc* 2011;12:964–73. doi: 10.1016/j.jpain.2011.03.003 21680251 PMC3222454

[52] Scherrer JF, Salas J, Copeland LA, et al. Prescription Opioid Duration, Dose, and Increased Risk of Depression in 3 Large Patient Populations. *Ann Fam Med* 2016;14:54–62. doi: 10.1370/afm.1885 26755784 PMC4709156

[53] Semenkovich K, Chockalingam R, Scherrer JF, et al. Prescription Opioid Analgesics Increase Risk of Major Depression: New Evidence, Plausible Neurobiological Mechanisms and Management to Achieve Depression Prophylaxis. *Mo Med* 2014;111:148–54. 30323529 PMC6179498

[54] Sheng J, Liu S, Wang Y, et al. The Link between Depression and Chronic Pain: Neural Mechanisms in the Brain. *Neural Plast* 2017;2017:9724371. doi: 10.1155/2017/9724371 28706741 PMC5494581

[55] Mental health disorders and chronic opioid use among adolescents and young adults with chronic pain—PMC. https://www.ncbi.nlm.nih.gov/pmc/articles/PMC3368381/ (accessed 19 Apr 2022).

[56] Feingold D, Brill S, Goor-Aryeh I, et al. The association between severity of depression and prescription opioid misuse among chronic pain patients with and without anxiety: A cross-sectional study. *J Affect Disord* 2018;235:293–302. doi: 10.1016/j.jad.2018.04.058 29660645

[57] Improving Opioid Prescribing | American Dental Association. https://www.ada.org/resources/practice/health-and-wellness/improving-opioid-prescribing (accessed 19 Apr 2022).

[58] Heron MJ, Nwokorie NA, O’Connor B, et al. Survey of opioid prescribing among dentists indicates need for more effective education regarding pain management. *J Am Dent Assoc 1939* 2022;153:110–9. doi: 10.1016/j.adaj.2021.07.018 34689958

[59] Calcaterra S, Glanz J, Binswanger IA. National trends in pharmaceutical opioid related overdose deaths compared to other substance related overdose deaths: 1999–2009. *Drug Alcohol Depend* 2013;131:263–70. doi: 10.1016/j.drugalcdep.2012.11.018 23294765 PMC3935414

[60] Darnall BD, Stacey BR, Chou R. Medical and Psychological Risks and Consequences of Long-Term Opioid Therapy in Women. *Pain Med Malden Mass* 2012;13:1181–211. doi: 10.1111/j.1526-4637.2012.01467.x 22905834 PMC4801003

[61] The Mental Status Examination—PubMed. https://pubmed.ncbi.nlm.nih.gov/21250162/ (accessed 19 Apr 2022).

[62] Simon L, Obadan-Udoh E, Yansane A-I, et al. Improving Oral-Systemic Healthcare through the Interoperability of Electronic Medical and Dental Records: An Exploratory Study. *Appl Clin Inform* 2019;10:367–76. doi: 10.1055/s-0039-1688832 31141831 PMC6541474

[63] Underlying Cause of Death, 1999–2020 Request. https://wonder.cdc.gov/ucd-icd10.html (accessed 19 Apr 2022).

[64] Gomes T, Mamdani MM, Dhalla IA, et al. Opioid Dose and Drug-Related Mortality in Patients With Nonmalignant Pain. *Arch Intern Med* 2011;171:686–91. doi: 10.1001/archinternmed.2011.117 21482846

[65] Benzodiazepine prescribing patterns and deaths from drug overdose among US veterans receiving opioid analgesics: case-cohort study | The BMJ. https://www.bmj.com/content/350/bmj.h2698 (accessed 19 Apr 2022). 26063215 10.1136/bmj.h2698 PMC4462713

[66] Ferreira G E, Abdel-Shaheed C, Underwood M, Finnerup N B, Day R O, McLachlan A et al. Efficacy, safety, and tolerability of antidepressants for pain in adults: overview of systematic reviews BMJ 2023; 380: e072415 doi: 10.1136/bmj-2022-072415 36725015 PMC9887507

[67] Stannard C, Wilkinson C. Rethinking use of medicines for chronic pain BMJ 2023; 380: p170 doi: 10.1136/bmj.p170 36724987

[68] Patel S, Carmichael JM, Taylor JM, et al. Evaluating the Impact of a Clinical Decision Support Tool to Reduce Chronic Opioid Dose and Decrease Risk Classification in a Veteran Population. *Ann Pharmacother* 2018;52:325–31. doi: 10.1177/1060028017739388 29086587

