# Supplementary material for: Opioids for acute and chronic pain when receiving psychiatric medications
Source: PLoS One. 2023 Sep 26;18(9):e0286179. doi: 10.1371/journal.pone.0286179 (PMC10522028; doi:10.1371/journal.pone.0286179)
Supplement: S1 Appendix — (DOCX) [file pone.0286179.s001.docx]

| **Appendix A. ICD Codes used to Categorize Patient Pain-related Classification** | |
| --- | --- |
| ***Diagnosis*** | ***ICD-9 CM codes*** |
| ENT (ear, nose, and throat) including otalgia | 388.70, 388.71, 388.72 |
| Dental related conditions | 520.0 – 529.9 (except 524.60, 524.64 & 524.69) |
| Orthopedics—strains, sprains, fractures | 810.00 to 819.00, 820.00 to 829.00, 840.00 to 848.00 |
| Back pain | 724.5 |
| Neck pain | 723.1 |
| Headache - cluster headaches, chronic headache and migraine pain | 339.00 339.01 339.02, 346.0 346.9 346.1, 346.7, 784.0 |
| Join pain - Osteoarthritis and Rheumatoid arthritis | 714.0, 715.0 to 715.98 |

| **Appendix B. Morphine Milligram Equivalents (MME) for Pain conditions by Antidepressant/Anxiety Medications** | | | | | |
| --- | --- | --- | --- | --- | --- |
| ***Morphine Milligram Equivalents (MME)*** | **00-19.9** | **20-49.9** | **50-89.9** | **90+** | **Total** |
| Patients with no history of Antidepressant/antianxiety medications | 250,925 (23%) | 690,269 (63%) | 116,019 (11%) | 39,966 (4%) | 1,097,179 (100%) |
| Patients with Antidepressant/antianxiety medications | 372,876 (22%) | 966,238 (57%) | 215,548 (13%) | 128,548 (8%) | 1,683,210 (100%) |
|  | | | | | |
| **MME Group distribution by Pain conditions** | | | | | |
| ***Morphine Milligram Equivalents (MME)*** | **00-19.9** | **20-49.9** | **50-89.9** | **90+** | **Total** |
| Acute Pain | 196,348 (20%) | 628,652 (64%) | 113,908 (12%) | 39,682 (4%) | 978,590 (100%) |
| Chronic Pain | 380,679 (24%) | 893,903 (56%) | 195,928 (12%) | 121,533 (8%) | 1,592,043 (100%) |
| Both Pain Types | 46,774 (22%) | 133,952 (64%) | 21,731 (10%) | 7,299 (3%) | 209,756 (100%) |
|  | | | | | |
| **MME Group Distribution for All Adult Opioid Patients 2012-2019** | | | | | |
| ***Morphine Milligram Equivalents (MME)*** | **00-19.9** | **20-49.9** | **50-89.9** | **90+** | **Total** |
| All Adult Opioid Patients | 7,145,956 (20%) | 19,970,000 (56%) | 5,123,915 (14%) | 3,499,804 (10%) | 35,737,558 (100%) |
